# *Aspergillus flavus* and *Fusarium verticillioides* Interaction: Modeling the Impact on Mycotoxin Production

**DOI:** 10.3389/fmicb.2019.02653

**Published:** 2019-11-12

**Authors:** Marco Camardo Leggieri, Paola Giorni, Amedeo Pietri, Paola Battilani

**Affiliations:** ^1^Department of Sustainable Crop Production, Università Cattolica del Sacro Cuore, Piacenza, Italy; ^2^Department of Animal Science, Food and Nutrition, Università Cattolica del Sacro Cuore, Piacenza, Italy

**Keywords:** mycotoxin, temperature, co-occurrence, aflatoxin, fumonisin

## Abstract

The influence of climate change on agricultural systems has been generally accepted as having a considerable impact on food security and safety. It is believed that the occurrence of mycotoxins will be greatly affected by future climate scenarios and this has been confirmed by recent data. Temperature (*T*) and CO_2_ increases, variation in rain intensity and distribution, as well as extreme weather events, affect the dominant fungal species in different ways, depending on their ecological needs. Therefore, the aim of this work was to study *Aspergillus flavus* (*Af*) and *Fusarium verticillioides* (*Fv*) co-occurrence *in vitro* in order to collect quantitative data on the effect of fungal interaction on growth and mycotoxin production and develop functions for their description. Experimental trials were organized with the cited fungi grown alone or together. They were incubated at different *T* regimes (10–40°C, step 5°C) for 21 days. Fungal growth was measured weekly, while AFs and FBs were quantified at the end of the incubation period. Temperature and incubation time significantly affected fungal growth both for *Af* and *Fv* (*p* ≤ 0.01), and a significant interaction between *T* and the presence of one *versus* both fungi influenced the amount of AFs and FBs produced. Each fungus was affected by the presence of the other fungus; in particular, *Af* and *Fv* showed a decrease in colony diameter of 10 and 44%, respectively, when they were grown together, compared to alone. The same influence was not found for mycotoxin production. In fact, the dynamics of toxin production in different temperature regimes followed a comparable trend with fungi grown alone or together, but a significant impact of inoculum × temperature interaction was highlighted. Fungal growth and toxin production in different *T* regimes were well described, both for AFs and FBs, by a Bete function. These results are the first attempt to model mycotoxigenic fungal co-occurrence under several *T* regimes; this is essential in order to improve effective prediction of growth and mycotoxin production by such fungi.

## Introduction

Mycotoxins contaminate the diet of a large proportion of the world’s population and represent a global public health issue (Wambacq et al., 2016), with the highest exposure reported in developing countries (Shephard, 2008). Young children and infants are particularly at risk and around three times more vulnerable than adults to the toxic effects of mycotoxins, because of their higher intake/body weight ratio, higher metabolic rate and lower detoxification capacity (Hulin et al., 2014).

Maize grain is a suitable host for several mycotoxin-producing fungi both in field and postharvest. In proper environmental and storage conditions, fungi present in maize grains may produce different mycotoxins, frequently co-occurring, which can induce toxic responses in humans and animals after ingestion (Grenier and Oswald, 2011; Queiroz et al., 2012). The primary mycotoxins occurring in maize worldwide are aflatoxins (AFs) and fumonisins (FBs) (Abbott, 2013; Rodrigues and Naehrer, 2013; Giorni et al., 2016), with *Aspergillus flavus* (*Af*) and *Fusarium verticillioides* (*Fv*) as main producers, respectively (Vaamonde et al., 2003; Rosa Junior et al., 2019). Considering the potential risk associated with the presence of a single mycotoxin, the co-occurrence of these two mycotoxins can cause additive/interactive effects and somehow modify their toxicity to humans and animals in a not well-defined manner (Abbès et al., 2016). Several publications have recently documented the co-occurrence of FBs and AFs in maize-growing areas where human hepatocellular carcinoma (HCC), chronic liver disease and growth retardation in children are consistently reported (Shirima et al., 2013; Probst et al., 2014). The combination of FBs and AFs is of particular concern because of the known genotoxicity of aflatoxin B_1_ (AFB_1_) and the ability of fumonisin B_1_ (FB_1_) to induce regenerative cell proliferation (Bulder et al., 2012). Awareness of human co-exposure to co-occurring mycotoxins is currently rising in several countries. In Tanzania, child growth impairment was found to be significantly associated with FB urinary levels; a relatively low aflatoxin exposure was also documented (Chen et al., 2018). Other co-occurring mycotoxins have been studied in Ecuador, such as ochratoxin A and deoxynivalenol (DON) in wheat-based products (Ortiz et al., 2018). In addition to its natural occurrence, mycotoxin co-occurrence can come from compound food. In fact, AFM_1_ and DON have been detected in products destined for infants and toddlers in India (Gummadidala et al., 2019); AF and DON have been found in cereal based baby food distributed in Europe (Herrera et al., 2019); fusarium toxins and OTA co-contaminated cereal-based infant/toddler food in the United States (Zhang et al., 2018). These studies confirm the alarming significance of co-occurring mycotoxins for human health, particularly for the high-risk population of babies, but also for the less considered group of toddlers.

Considering the global occurrence of mycotoxins, with 72% of samples analyzed worldwide resulting positive (Schatzmayr and Streit, 2013), their crucial role in human and animal health as the greatest cause of chronic foodborne disease (Kuiper-Goodman, 2004) and the economic losses resulting from over-contaminated maize (Oliveira et al., 2017), efforts have to be addressed to fill the lack of knowledge and contribute to mycotoxin mitigation. In particular, extreme weather events are expected to be more frequent due to climate change, with a strong impact on mycotoxins (Miraglia et al., 2009; Huber and Gulledge, 2011; IPCC, 2012; Battilani and Logrieco, 2014).

*Af* is prevalent in tropical areas, but its occurrence in Europe has increased since the 2000s, particularly during dry and warm summers (Kos et al., 2013). Moreover, in 2003 the first European outbreak of AFs was reported in northern Italy (Piva et al., 2006; Battilani et al., 2008), and in 2012 outbreaks were reported in south eastern Europe (Dobolyi et al., 2013; Levic et al., 2013; De Rijk et al., 2015), events attributed to climate change (Battilani et al., 2016). On the other hand, FB-producing fungi can be found wherever maize is grown (Miller, 2001; Bush et al., 2004; Wu et al., 2011).

Very variable weather conditions, even during the growing season, are likely to both favor fungi with very different ecological needs and to enhance fungi and mycotoxin co-occurrence (Dall’Asta and Battilani, 2016; Obradovic et al., 2018; Camardo Leggieri et al., submitted). We are headed toward a changing world and, in this context, as stressed by Vaugham et al. (2016), modeling approaches that combine data on climate, pathogen and host, including cropping systems, could provide great support to the value chain management by predicting mycotoxin risk under the anticipated scenarios (Battilani, 2016). Predictive models for aflatoxin (AFLA-maize; Battilani et al., 2013) and fumonisin (FER-maize; Battilani et al., 2004) contamination in maize are available, but the two models can only be run separately, and they do not account for interactions among mycotoxin-producing fungi.

Therefore, the aim of this study was to: i) acquire knowledge regarding the interaction of *Af* and *Fv*, commonly co-occurring in maize, in different ecological conditions; ii) quantify the impact of interaction on fungal growth and mycotoxin production; iii) implement mathematical functions accounting for the impact of fungi interaction on growth and toxin production, to be included in predictive models and develop a joint predictive model for AFs and FBs.

## Materials and Methods

### Experiment Description

The interaction between *Af* and *Fv* was studied *in vitro*, in different culture conditions, in order to quantify the impact of fungal co-occurrence on their growth and mycotoxin production.

One strain of *Af* (ITEM 8069) and 1 strain of *Fv* (ITEM 10027) able to produce, respectively, aflatoxin B_1_ and B_2_ and fumonisin B_1_, B_2_, B_3_, stored in the official fungal collection of the Institute of Sciences of Food Production of the National Research Council (ISPA-CNR) in Bari, were used for inoculum preparation. The isolates were inoculated on the surface of Potato Dextrose Agar (PDA, Biolife, Milano, Italy) in Petri dishes and incubated at 25°C for 7 days (12 h light/12 h dark photoperiod). At the end of incubation, developed fungal colonies were used as inoculum source for the experiments.

Maize flour, free from mycotoxins, was recovered by food producers and used for preparing artificial maize medium (maize flour:water 1:2.5). The medium was tyndallized (instead of sterize, to minimize heat effect on medium composition) by heating for 30 min at 80°C and cooling down three times (Lazzaro et al., 2013). The corn meal medium (CMM) obtained was then poured into Ø 90 mm Petri dishes and stored at 5°C till used.

Inoculum for growth assays was prepared by growing the *Af* and *Fv* strains axenically on PDA. Five-mm-diameter pieces of the resulting cultures were then used to inoculate the surface of CMM and PDA media contained in 90 mm Petri dishes. When the fungi were grown alone, an inoculum piece was placed on the medium surface in the center of the Petri dish. When the fungi were grown together, an inoculum piece for each fungus was placed on the medium surface along a diameter so that the distances between the inoculum pieces and the edges of the dish were the same.

Inoculated Petri dishes were incubated at different temperature (*T*), from 10 to 40°C, with 5°C increments (12 h light/12 h dark photoperiod) and fungal growth was measured (two perpendicular diameters of the fungal colony) at different times of incubation: 3, 7, 10, 14, and 21 days. The experiments were conducted twice, each time in triplicate.

At the end of incubation (21 days), the entire content of the CMM Petri dishes was used for mycotoxin analysis. Samples were dried at 65°C for 2 days, milled and homogenized before analysis. Sample preparation, extraction and analyses were performed according to the methods reported by Bertuzzi et al. (2012) for AFs, Pietri and Bertuzzi (2012) for FBs. Briefly, AFB_1_, AFB_2_, AFG_1_, and AFG_2_ were determined using an HPLC instrument with a fluorescence detector; FB_1_ and FB_2_ were determined using an HPLC-MS/MS system. Results were reported as μg of mycotoxin per kg of CMM.

The limit of detection (LOD) and quantification (LOQ) were, respectively: 0.05 and 0.15 μg/kg for each AF, 10 and 30 μg/kg for each FB.

### Data Analysis

Data analysis was done using IBM SPSS Statistics 24 (SPSS Inc., Chicago, IL, United States). Fungal growth was calculated as mean growth on PDA and CMM media, while mycotoxin production was measured only on CMM medium.

All the data obtained were subjected to univariate analysis of variance (ANOVA) using the generalized linear model (GLM) procedure and significant differences between means were confirmed using the Tukey test. In particular, for fungal growth data, the main effects “inoculum thesis” (2 levels), “temperature” (7 levels), “time of incubation” (5 levels), and “medium” (2 levels) were tested as independent variables, as well their interactions. Likewise, for mycotoxin production, the same main effects were considered, except “time of incubation.” All mycotoxin production data were transformed by *y* = ln(*x*) before ANOVA analysis to homogenize the variance.

In order to model fungal growth and mycotoxin production, data on *Af* and *Fv* grown alone were rated on the maximum value observed to obtain growth/mycotoxin rate on a 0–1 scale, with 0 = no growth/no mycotoxin production, and 1 = maximum growth/toxin production. Data collected when fungi were grown together were rated on the maximum value observed when each fungus was grown alone to quantify the impact on growth/toxin production due to fungal interaction.

The non-linear regression model of Bete-Analytis (Analytis, 1977) was fitted to the collected data in order to describe fungal growth and mycotoxin production as function of *T*; the function was chosen based on the good performances obtained in previous studies (Camardo Leggieri et al., 2017, 2018).

The equation applied follows:

(1) $$\begin{array}{c} y_{T}={\left(a\times {\left(T\,\text{eq}\right)}^{b}\times \left(1-T\,\text{eq}\right)\right)}^{c}\\ T\,\text{eq}=\left(\frac{T-T\,\text{min}}{T\,\text{max}-T\,\text{min}}\right) \end{array}$$

where *T*eq is an equivalent *T* fixing the limits for growth/mycotoxin production, *T*min is minimum *T*, *T*max is maximum *T*, *a* and *c* are the equation parameters accounting for the height and width of the bell-shaped curve, respectively, while *b* determines the *T* values at which the curve reaches the maximum.

The equation parameters were estimated applying the non-linear regression procedure of IBM SPSS Statistics, which minimizes the residual sum squares error using the Levenberg–Marquardt algorithm.

## Results

All the experiments were performed twice, and the data obtained from replicate experiments were not significantly different (data not shown). Therefore, data from replicate experiments were analyzed together.

### Fungal Growth

The ANOVA was applied to all data on fungal growth in Table 1. Treatment applied (*Af* and *Fv* grown alone or together), *T* and incubation time, significantly affected fungal growth, both for *Af* and *Fv* (*p* ≤ 0.01), while the growth medium only impacted significantly on *Fv* (Table 1). *Af* growth was significantly affected when *Fv* was grown together (*p* ≤ 0.05); a 10% decrease was observed in *Af* colony diameter with fungi grown together versus fungus grown alone.

**TABLE 1:** Analysis of variance (ANOVA) of *Aspergillus flavus* (*Af*) – *Fusarium verticillioides* (*Fv*) growth (mm) and aflatoxin B_1_ (AFB_1_) and fumonisin B_1_ + B_2_ (FBs) contamination (μg/kg) in the different treatments considered (fungi grown alone or together), temperature (10–40°C, step 5°C), time of incubation (3, 7, 10, 14, and 21 days) and medium (CMM or PDA).

	**Growth (mm)**				**Mycotoxin (μg/kg)**			
		
	***Af***		***Fv***		***AFB*_1_**		***FBs***	
*Treatment*	*^∗^*		*^∗∗^*		*n.s.*		*n.s.*	
Alone	38.8	*a*	27.2	*a*	32,487		92,658	
Together	35.3	*b*	11.9	*b*	25,191		57,981	
*Temperature (°C)*	^∗∗^		^∗∗^		^∗∗^		^∗∗^	
10	0.0	*f*	0.1	*e*	6	*d*	0	*d*
15	26.7	*d*	35.2	*b*	28,175	*b*	30,929	*ab*
20	49.7	*c*	39.3	*a*	62,013	*a*	258,706	*a*
25	54.0	*bc*	37.4	*ab*	60,740	*a*	199,220	*a*
30	72.9	*a*	23.3	*c*	20,168	*c*	20,081	*b*
35	58.7	*b*	12.1	*d*	1,470	*d*	157	*c*
40	13.1	*e*	0.0	*e*	0	*d*	0	*d*
*Time of incubation (days)*	^∗∗^		^∗∗^					
3	23.4	*c*	12.4	*c*				
7	29.2	*c*	16.8	*b*				
10	40.4	*b*	15.3	*b*				
14	43.0	*ab*	23.9	*a*				
21	47.6	*a*	25.5	*a*				
*Medium*	n.s.		^∗∗^					
CMM	41.6		14.4	*b*				
PDA	32.6		24.7	*a*				

*n.s.: non-significant; ^∗^*p* ≤ 0.05; ^∗∗^*p* ≤ 0.01.*

**FIGURE 1:**
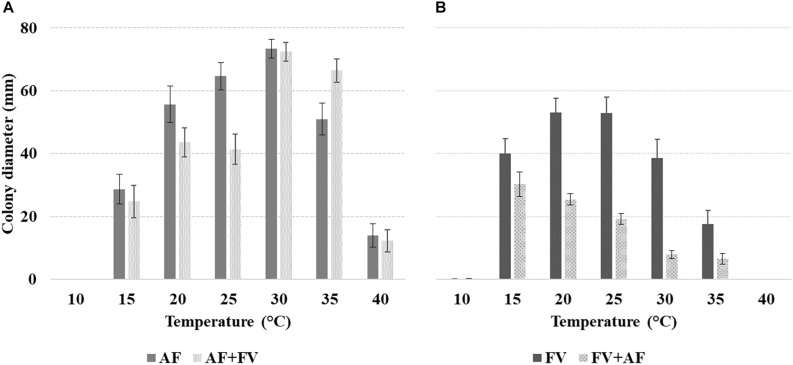
Mean colony diameter (mm) of alone fungus inoculum of **(A)** *Aspergillus flavus* (*Af*) and together fungi inoculum of *Af* + *Fv* and **(B)** *Fusarium verticillioides* (*Fv*) and together fungi inoculum of *Fv* + *Af*, at different *T* of incubation (10–40°C, 5°C step) on both media considered (CMM and PDA). The bars indicate the mean standard error. All experiments were conducted using three replicates and were performed twice.

*Af* growth was significantly affected by incubation *T*; growth was appreciable from 15 to 40°C, with maximum colony diameter reached at 30°C (72.9 mm) (Figure 1A). The impact of *T* increase was stronger compared to *T* decrease from the optimal condition; colony diameter was 80% reduced at 40°C *versus* 30% at 20°C, compared to 30°C.

**FIGURE 2:**
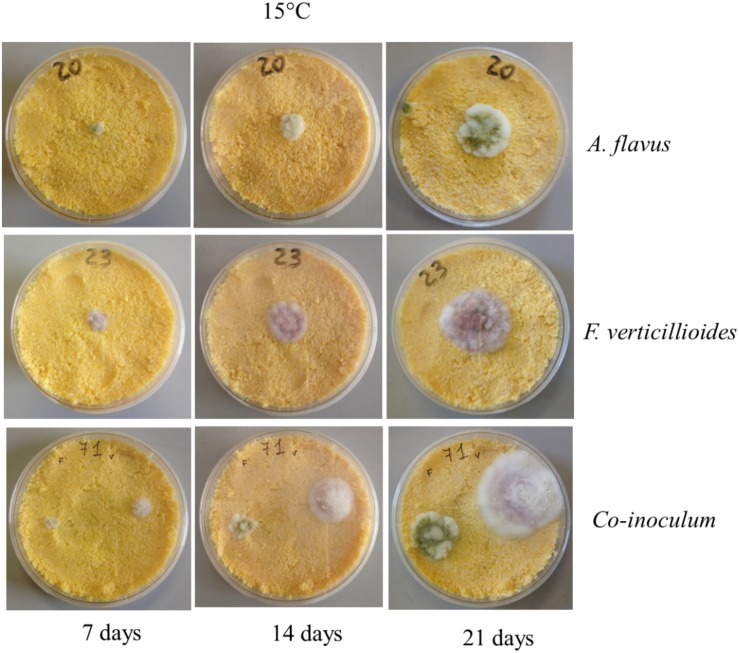
Example of *Aspergillus flavus* (*Af*) and *Fusarium verticillioides* (*Fv*) growth with alone colonies and together colonies on corn meal medium (CMM) incubated at 15°C for 7, 14, and 21 days.

As expected, incubation time also significantly affected *Af* growth; colonies were visible after 3 days of incubation, when their diameter was around half the maximum, reached after 21 days of incubation (Figure 2). No significant effect was observed when *Af* was grown on PDA or CMM (Table 1).

Some interactions between factors were significant; of particular interest is the interaction between alone fungus/together fungi growth and incubation *T*. At 20 and 25°C *Af* growth was affected by the co-inoculum of *Fv* and colony diameter was significantly lower compared to that measured in alone colonies; on the contrary, at 35°C *Af* growth was enhanced by the presence of *Fv* (Figure 1A).

All the tested factors and their interactions had a significant impact (*p* ≤ 0.01) on *Fv* growth. Forty-four percent colony diameter decrease was observed when *Fv* was grown together with *Af versus Fv* grown alone. *Fv* was unable to grow with *T* ≥ 40°C, minimal growth at 10°C, and optimal growth at 20–25°C (mean diameter 38 mm) (Figure 1B). A significant decrease in colony diameter was observed moving to 30°C (−64%) and 35°C (−68%).

As expected, also for *Fv* the incubation time had a significant effect on colony growth (*p* ≤ 0.01); the colony was visible after 3 days and doubled in size after 14 days of incubation. The different media used had a significant impact; on the CMM *Fv* colony diameter was 55% smaller than on PDA (Table 1).

The interaction fungi grown alone/together and incubation *T* were significant also for *Fv*; colony growth was strongly reduced, at all *T* regimes, by the presence of *Af* and maximum colony growth when fungi were grown together was observed at 15°C. The highest decrease in colony diameter with fungi grown together *versus Fv* alone (−65%) was observed at 25°C (Figure 1).

### Mycotoxin Production

The ANOVA was applied to all data on mycotoxins produced after 21 days of incubation on CMM medium (Table 1). As regards AFs, only AFB_1_ was considered, because the production of AFB_2_, AFG_1_ and AFG_2_ was negligible with respect to AFB_1_. For FBs, the sum of FB_1_ and FB_2_ was calculated and used in data processing. Treatment applied, intended as *Af* and *Fv* grown alone or together, did not affect significantly mycotoxin production; on the contrary, *T* had a significant impact, both for AFB_1_ and FBs (*p* ≤ 0.01), as did treatment × *T* interaction.

**FIGURE 3:**
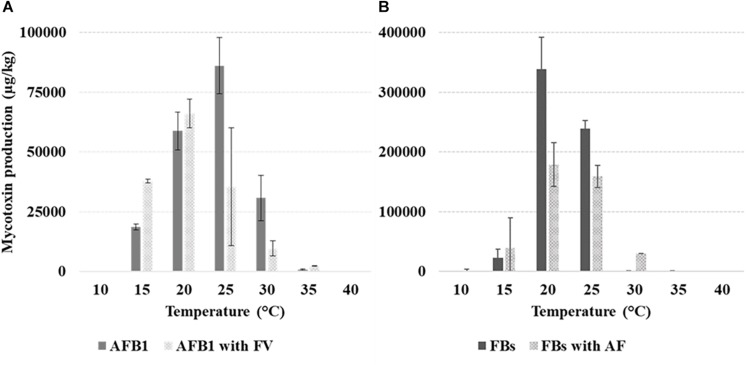
Production (μg/kg) of aflatoxin B_1_ (AFB_1_) **(A)** and fumonisins [FB_1_ + FB_2_ (FBs)] **(B)** by *Aspergillus flavus* (*Af*) and *Fusarium verticillioides* (*Fv*) grown alone or together, at different *T* of incubation (10–40°C, 5°C step). The bars indicate the mean standard error. All experiments were conducted using three replicates and were performed twice.

Mean AFB_1_ production in colonies of *Af* when grown alone was 32,487 μg/kg versus 25,191 μg/kg in presence of *Fv*. AFB_1_ was produced from 10°C, with the optimum observed, without significant differences, at 20–25°C. At 15°C, the production was greatly and significantly reduced as well as with *T* ≥ 30°C (Table 1). The interaction treatment × *T* showed a significantly higher AFB_1_ production at 15 and 20°C significantly lower at 25 and 30°C with *Fv* grown together with *Af* compared to *Af* grown alone (Figure 3).

FB production was 92658 μg/kg *versus* 57981 μg/kg with *Fv* grown alone and together with *Af*, respectively. FBs were produced from 15 to 35°C, the highest level was reported at 20–25°C, significantly different from the production at 30 and 35°C. The interaction treatment × *T* showed a significantly higher FB production with *Fv* grown alone *versus* together with *Af* at 20–25°C.

### Modeling the Role of *T* on Fungal Growth

**FIGURE 4:**
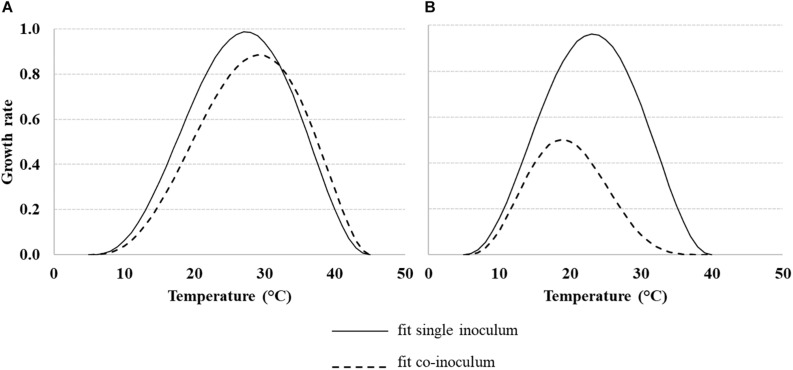
Dynamic of the growth rate of *Aspergillus flavus* (*Af*) **(A)** and *Fusarium verticillioides* (*Fv*) **(B)**, under different temperature regimes (10–40°C), grown alone or together. Data were fitted by a non-liner function (Eq. 1, Table 2 for equation parameters) both for fungi grown alone (solid line) and together (dotted line).

For modeling the growth rate of *Af*, *Fv* and their interaction (*Af* + *Fv* and *Fv* + *Af*) Eq. 1 was used; the dynamic in different *T* regimes is represented in Figure 4. Standard errors of the estimated parameters were around 10 times lower than the parameter itself (Table 2), confirming the goodness of fit of the applied equation.

**TABLE 2:** Estimated parameters (*a*, *b*, and *c*) and summary statistics (standard errors of parameters were reported in parenthesis) of non-linear regression analysis (Eq. 1) developed to calculate the growth rate of *Aspergillus flavus* (*Af*) (alone, *Af* or together, *Af* + *Fv*) and *Fusarium verticillioides* (*Fv*) (alone, *Fv* or together, *Fv* + *Af*) as function of temperature (*T*).

	**Teq**	**Parameters**			***R*^2^**
			
		***a***	***b***	***c***	
*Af*	5–45	4.70	1.26	2.30	0.90
		(0.298)	(0.095)	(0.441)	
*Af* + *Fv*	5–45	5.15	1.59	1.93	0.81
		(0.539)	(0.179)	(0.544)	
*Fv*	5–40	4.15	1.08	2.23	0.83
		(0.369)	(0.127)	(0.688)	
*Fv* + *Af*	5–40	2.64	0.66	4.85	0.82
		(0.205)	(0.062)	(1.328)	

Solid line in Figure 4 represent the trend of *Af* and *Fv* grown alone, with shifted optimal *T*, 25–30°C and 20–25°C for *Af* (Figure 4A) and *Fv* (Figure 4B), respectively.

Dotted lines represent the growth rate of *Af* (Figure 4A) and *Fv* (Figure 4B) grown together; *Fv* had a minor impact on *Af*, while *Af* impact on *Fv* was quite strong. In fact, *Af* growth was slightly reduced by the presence of *Fv*, with a maximum growth rate ≈ 0.90, observed at 30°C. On the other hand, *Fv* maximum growth rate together (dotted line, Figure 4B) with *Af* was ≈ 0.50 (observed at 20°C).

### Modeling the Role of *T* on Mycotoxin Production

The same approach used for fungal growth was applied to model mycotoxin production rate over different *T* regimes (10–40°C) for AFB_1_ produced by *Af*, FBs produced by *Fv* and both toxins when fungi were grown together.

**FIGURE 5:**
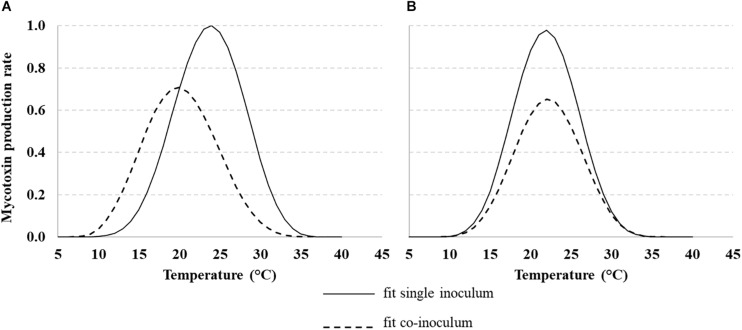
Dynamic of aflatoxin B1 (AFB_1_) **(A)** and fumonisins [FB1 + FB2 (FBs)] **(B)** production rate with *Aspergillus flavus* (*Af*) and *Fusarium verticillioides* (*Fv*) grown (solid line) or together (dotted line) under different *T* regimes (10–40°C). Data were fitted by a non-liner function (Eq. 1, Table 3 for equation parameters).

Mycotoxin production rates were fitted using Eq. 1 and the parameters are reported in Table 3. The AFB_1_ production rate was affected by *Af* co-occurring with *Fv* (Figure 5A, dotted line); the rate was lower when fungi were grown together and the optimum showed a shift from 25°C to 20°C. Regarding FBs, when *Fv* was grown together with *Af*, the production rate decreased, but the optimum was confirmed at 20°C (Figure 5B, dotted line).

**TABLE 3:** Estimated parameters and summary statistics (standard errors of parameters are reported in parenthesis) of non-linear regression analysis (Eq. 1) developed to calculate mycotoxin production rate (aflatoxin B_1_ for *Af* and fumonisin B_1_ and B_2_ for *Fv*) of alone and together fungi as function of temperature (*T*).

	**Teq**	**Parameters**			***R*^2^**
			
		***a***	***b***	***c***	
*Af*	5–45	4.47	1.17	6.71	0.99
		(0.090)	(0.031)	(0.698)	
*Af* + *Fv*	5–45	3.11	0.74	7.26	0.99
		(0.053)	(0.018)	(0.642)	
*Fv*	5–45	3.79	0.93	9.16	0.96
		(0.132)	(0.044)	(1.446)	
*Fv* + *Af*	5–45	3.69	0.96	8.37	0.98
		(0.120)	(0.040)	(0.897)	

## Discussion

This study examined, for the first time, the effect of a wide range of temperatures (from 10 to 40°C) on *Af* and *Fv* growth and mycotoxin production when the fungi were grown together on laboratory media. The occurrence of these two fungi in maize is important because they are able to produce AFs and FBs, the two most important groups of mycotoxins detected in this crop worldwide (Herteg et al., 2016; Shu et al., 2017; Obradovic et al., 2018). In Europe, the presence of both mycotoxins in maize destined for human and animal consumption is regulated (European Commission [EC], 2003, 2006, 2007, 2010). Recently, attention has been focused on some cohort populations, for example toddlers and the elderly, who are sensitive to mycotoxins similarly to babies, but who are not protected by specific regulations (Chen et al., 2018; Papageorgiou et al., 2018; Valitutti et al., 2018; Gummadidala et al., 2019). This further stresses the relevance of mycotoxin co-occurrence and the importance of acquiring knowledge for co-occurrence prediction.

At each *T* considered, fungal growth with the two fungi together *versus* alone were compared. The behavior of *Af* and *Fv* grown alone was comparable to those obtained in previous *in vitro* studies. The optimal *T* for AF production by *Af* was 30°C, whereas a previously reported optimum was 28°C (Sanchis and Magan, 2004). Likewise, we found the optimal *T* for FB production by *Fv* to be 20–25°C, whereas the previously reported optimum was 25–30°C (Marín et al., 1995; Giorni et al., 2009). However, the interaction of fungi that produce different mycotoxins has scarcely been considered in literature (Marín et al., 1998a, b; Giorni et al., 2009); the competition of fungal species for nutritional sources under different environmental conditions was considered, but without quantifying the impact of fungal interaction.

The effect of co-culturing *Af* and *Fv* on the growth of *Af* varied with temperature. Growth of *Af* in co-cultures was reduced at 15–25°C, unaffected at 30°C, and increased at 35°C compared to when *Af* was grown alone. *Af* had a stronger impact on *Fv* growth compared to the opposite; in fact, *Fv* colony diameter was always decreased by the presence of *Af*. This stronger impact of *Af* on *Fv*, compared to the opposite, is in agreement with a recent study conducted in field where *Af* incidence was reduced by 10% in the presence of *Fv*, while *Fv* showed a 44% reduction in incidence in the case of *Af* co-occurrence (Giorni et al., 2019). This is probably due to the different efficiency and rapidity of the two fungi to use carbon sources and invade the substrate. In fact, as already demonstrated in other studies, *Af* used carbon sources more rapidly than *Fv* at high *T* (*T* = 25–30°C) and dry conditions (0.87a_w_); Instead, *Fv* was dominant at 15°C, the lowest *T* tested in the aforementioned study (Giorni et al., 2009), being able to use more carbon sources. In addition, at 15°C the colony diameter of *Fv* was greater than *Af*, in agreement with an experiment reported by Marín et al. (1998a), where the infection of maize kernels by *Fusarium* spp. at 25°C was strongly influenced by the co-occurrence of *Af* and *Aspergillus niger*, with a reduction of kernel infection up to 45 and 30%, respectively, after 14 days of incubation.

Co-culturing *Af* and *Fv* affected mycotoxin production, again influenced by the temperature regime; in particular, when fungi are grown together, AFB_1_ production increased at 15–20°C while FB production decreased at 20–25°C. An increment in AFB_1_ production was observed at 20°C when *Af* growth was greatly reduced by *Fv* co-occurrence, while FB production was reduced when *Fv* was at its optimal *T* for growth (*T* ≥ 20°C). Therefore, it seems confirmed that mycotoxin production is highly dependent on fungal stress induced by both unfavorable environmental conditions and, probably, competition due to the co-occurrence of fungi in the same substrate. Apparently, *Fv* causes more stress to *Af* in suboptimal *T* conditions than the opposite, enhancing toxin production; as previously suggested by Schmidt-Heydt et al. (2008) under certain environmental stress conditions there is a stimulation of toxin production, as stated by gene response.

No statistically significant differences were found between AFB_1_ and FBs produced with the producing fungi grown alone or together, but the interaction treatment × *T* caused a significant impact. This was partially in agreement with findings of the previously mentioned study conducted on maize ears in field (Giorni et al., 2019), where only AFB_1_ was unvaried while FBs were partially reduced in the case of fungi co-occurrence. However, this apparent discrepancy could be due to *in vitro versus in vivo* conditions (artificial medium *versus* maize kernels in growing plants), including the role of weather conditions. In field experiments in which maize ears were inoculated with *Fv* and *Fusarium graminearum* (*Fg*), either alone or together, *Fv* outcompeted *Fg* (Picot et al., 2012). In some cases, *Fv* was able to outcompete *Fg* even when the *Fg* inoculation was done a week before the *Fv* inoculation.

The relevance of *T* for fungal occurrence is well known and is crucial for predictive modeling in several crops (Grinn-Gofroń and Strzelczak, 2013; Dummel et al., 2015; Khajuria Razdan and Atul Kumar, 2017). This important impact was stressed in the case of fungi grown together in this study and well described by the Analytic function. Equations describing the rate of each step of the fungal infection cycle are the core of mechanistic models (Madden et al., 2007). Several empiric/mechanistic models have been developed for mycotoxin prediction in maize (Blandino et al., 2009; Asselt et al., 2012; Chauhan et al., 2015). Two mechanistic models are currently in use for the prediction of FBs (Battilani et al., 2004) and AFB_1_ (Battilani et al., 2013) in maize and a worsening in their performances in Italy has been noticed recently. None of the models considers the impact of fungal co-occurrence, while fungi interaction could play a crucial role in climate change. Therefore, an update of predictive models would seem to be an emerging need.

Even with the limitations of this study, based on the use of one strain for each species, the implementation of new functions resulting from this work, taking into account fungi interaction and the influence of weather conditions, should have a positive impact on model prediction reliability. The variability between fungal strains in mycotoxin production, in different ecological regimes, has been little reported in literature and few strains have been included in the studies. To mentioning a couple of examples: *Fv* strains did not show a significant impact on FB production rates and *FUM* gene expression when two strains were compared (Lazzaro et al., 2012). Regarding *A. flavus*, a recent work underlined the differences in aflatoxin gene clusters, resulting in differences in toxin production, only between L or S strains (Gilbert et al., 2018), two groups with clear phenotypic differences. Further, for the development of FER-maize and AFLA-maize, the predictive models for FBs (Battilani et al., 2004) and AFB_1_ (Battilani et al., 2013) in maize, data collected using different fungal strains, deriving from different papers, were consistent and successfully used for model function development.

## Conclusion

This study represents a step forward for the emerging topic of co-occurring fungi in maize, but due to the complexity of fungus-plant-environment interactions, additional *in vitro* studies should be conducted to further refine understanding on how the interaction of different species of mycotoxin-producing fungi impact on mycotoxin production; these studies must be combined with *in planta* experiments, to confirm the resulting contamination in crops and to support updated predictive model validation.

## Data Availability Statement

The datasets generated for this study are available on request to the corresponding author.

## Author Contributions

MC, PG, and PB conceived and designed the experiments. PG performed the experiments. AP performed the mycotoxin analysis. MC and PB analyzed the data. All authors significantly contributed with writing, reviewing, and editing the manuscript.

## Conflict of Interest

The authors declare that the research was conducted in the absence of any commercial or financial relationships that could be construed as a potential conflict of interest.
